# INDRA-IPM: interactive pathway modeling using natural language with automated assembly

**DOI:** 10.1093/bioinformatics/btz289

**Published:** 2019-05-09

**Authors:** Petar V Todorov, Benjamin M Gyori, John A Bachman, Peter K Sorger

**Affiliations:** Laboratory of Systems Pharmacology, Harvard Medical School, Boston, MA, USA

## Abstract

**Summary:**

INDRA-IPM (Interactive Pathway Map) is a web-based pathway map modeling tool that combines natural language processing with automated model assembly and visualization. INDRA-IPM contextualizes models with expression data and exports them to standard formats.

**Availability and implementation:**

INDRA-IPM is available at: http://pathwaymap.indra.bio. Source code is available at http://github.com/sorgerlab/indra_pathway_map. The underlying web service API is available at http://api.indra.bio:8000.

**Supplementary information:**

Supplementary data are available at *Bioinformatics* online.

## 1 Introduction

Disease or process-specific pathway maps are commonly used to communicate mechanistic information about interacting genes and proteins (Ostaszewski *et al.*, 2018). These maps contain information on biomolecules and their interactions organized around a specific biological process, for instance growth-factor signaling mediated by RAS and MAP kinases (Stephen *et al.*, 2014). Unlike genome-wide interactomes, pathway maps are typically restricted in scope and are fit to purpose to improve human intelligibility and avoid the ‘hairball effect’.

Multiple graphical editing tools have been developed to assemble and display pathway maps (King *et al.*, 2015; O’Hara *et al.*, 2016; Sari *et al.*, 2015) but these do not currently use the primary medium of scientific communication in biomedicine: natural language. Natural language descriptions are familiar, do not require specialized expertise to create and edit, and can be drawn directly from the scientific literature (Gyori *et al.*, 2017). The use of natural language interfaces for pathway modeling and analysis makes it possible to draw on a much larger community of experts.

In this article, we describe the INDRA (Integrated Network and Dynamical Reasoning Assembler) Interactive Pathway Map (INDRA-IPM), a web-based pathway modeling tool that builds on the capabilities of INDRA (Gyori *et al.*, 2017) to construct and edit pathway maps in natural language and display the results in familiar graphical formats. INDRA-IPM allows models to be exported in several different standard exchange formats, thereby enabling the use of existing tools for causal inference, visualization and kinetic modeling. We also make the capabilities of IPM available as a web service to facilitate use by other software.

## 2 Results

### 2.1 Pathway map construction

INDRA-IPM provides an interface to enter English language text describing mechanisms used to generate a pathway map. This description is processed by one or more natural language processing (NLP) systems; users can choose between the REACH (Valenzuela-Escarcega *et al.*, 2017) and TRIPS (Allen *et al.*, 2015) NLP systems. A pathway map is then generated automatically from the NLP output and visualized dynamically. Users can iteratively update and extend the pathway map by editing the underlying natural language.

### 2.2 Visual representation

**Fig. 1.**
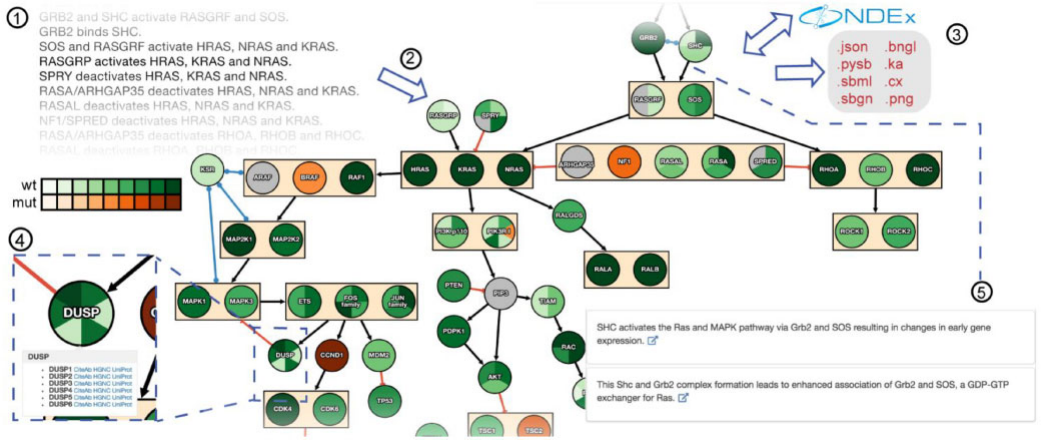
Pathway maps are assembled from natural language descriptions of mechanisms (1). INDRA-IPM renders pathway maps as graphs with node and edge grouping and coloring determined by mutational status and expression level (2). Pathways can be stored and shared on NDEx and exported and downloaded in many standard formats (3). Node tooltips provide links to online databases having information on genes/protein in the pathway and also to antibodies against proteins in the node (4). Literature-based evidence for a given interaction can be accessed by clicking on an edge. Corresponding evidence sentences drawn from these publications are then shown with links out to the PubMed entry in which the sentences are found (5)

The pathway map is represented as a directed graph with nodes corresponding to molecular entities (genes/proteins, families, complexes and small molecules) and edges representing mechanistic relationships among them. The graph is displayed using CytoscapeJS (Franz *et al.*, 2015) with a two-stage hierarchical layout procedure designed to reduce visual complexity. INDRA-IPM groups nodes with identical incoming and outgoing edges and aggregates them into a single bounding box with collapsed edges (e.g. RASGRF and SOS in Fig. 1). Nodes representing protein families or complexes (e.g. the Sprouty family, SPRY in Fig. 1), are recognized using the FamPlex ontology (Bachman *et al.*, 2018) and represented by a single node subdivided to show the genes in the family as slices.

### 2.3 Integration with modeling and exchange formats

To leverage automated assembly for diverse modeling tasks, INDRA-IPM exports models as SBML, SBGN, BNGL, Kappa, PySB and CX. These formats are widely used in computational biology for modeling, simulating and visualizing pathways. Users also have the option of storing maps on the Network Data Exchange (NDEx) (Pratt *et al.*, 2015) where they can be shared and reloaded into INDRA-IPM using a persistent URL. More details on these formats are available in the Supplementary Material.

### 2.4 Integration with gene level data

INDRA-IPM enables users to project mutation and expression data onto the pathway map and thereby visualize data specific to a particular cell type. Mutation status is mapped to color (with green nodes denoting wild-type and orange nodes mutations) and relative expression levels to color intensity (greater color saturation denotes higher expression). Cancer Cell Line Encyclopedia (CCLE; Barretina *et al.*, 2012) data are embedded in INDRA-IPM making it possible to view mutation and expression information for 996 cell lines.

### 2.5 Integration with external resources

The NLP tools used by INDRA-IPM link each node (or subnode in a family) to a database identifier using named entity recognition. This makes it possible to connect a pathway map to standard external resources via uniform identifiers. For example, by clicking on a node, a tooltip appears with links to HGNC, UniProt and CiteAb, allowing users to access details about the constituents of a pathway and identify reagents useful for experiments (e.g. antibodies).

### 2.6 Integration with evidence from scientific literature

Clicking on an edge in a pathway map retrieves support for that interaction by querying a database of interactions aggregated by INDRA. This database includes information gathered from reading literature at scale (Valenzuela-Escarcega *et al.*, 2017) and information found in curated knowledge bases such as Pathway Commons (Cerami *et al.*, 2011) and the BEL Large Corpus (www.openbel.org). Users are therefore able to access literature support for relationships specified in natural language descriptions.

### 2.7 RAS pathway map

As a demonstration of INDRA-IPM, we wrote 43 English sentences to capture all nodes and interactions in a pathway map originally created by the NCI RAS Initiative (cancer.gov/research/key-initiatives/ras). The INDRA-IPM map automatically follows the same visual conventions as the hand-drawn map, hierarchically organizing the graph and spatially grouping related nodes to reduce clutter. In addition, INDRA-IPM substantially extends the original map by providing access to supporting evidence sourced by INDRA, linking elements to external data resources and provide context from CCLE data. The RAS pathway map is available as a built-in example in INDRA-IPM.

### 2.8 Web service API

To facilitate integration of INDRA-IPM with other tools, we make it available a Web-based API that accesses reading, assembly and export functions of INDRA-IPM. 

## Funding

This work was funded under the DARPA Big Mechanism and CwC Programs [W911NF-14-1-0397 and W911NF-15-1-0544] and by NIH [P50-GM107618].

*Conflict of Interest*: P.K.S. holds equity in Merrimack Pharmaceuticals, Glencoe Software, Applied Biomath and RareCyte Inc. P.K.S. declares that none of these relationships are directly or indirectly related to the content of this article.

## Supplementary Material

btz289_Supplementary_Data
