# Supplementary material for: INDRA-IPM: interactive pathway modeling using natural language with automated assembly
Source: Bioinformatics. 2019 May 9;35(21):4501–3. doi: 10.1093/bioinformatics/btz289 (PMC6821420; doi:10.1093/bioinformatics/btz289)
Supplement: btz289_Supplementary_Data [file btz289_supplementary_data.pdf]

Supplementary Documentation for INDRA Interactive Pathway Map (INDRA-IPM)

Introduction

This website (http://pathwaymap.indra.bio) implements an interactive pathway map (IPM) built using INDRA, an automated model assembly system for molecular biology. The goal of INDRA-IPM is to allow users to build, contextualize, and share biological pathway models by describing them in natural language.

The visualization aims to display pathways in a visual style similar to that used by biologists in textbooks and presentations. In addition, we offer a layer of contextualization and an interactive user interface:

- Nodes represent biological entities mentioned in text.
- Nodes representing protein families or complexes are subdivided into pie charts based on their constituents.
- Wild-type genes are colored green.
- Mutated genes are colored orange.
- The intensity of each color corresponds to the expression level of a gene in CCLE.
- Clicking any node provides additional context by linking out to CiteAb, HGNC, and UniProt (for genes/proteins), and other resources such as ChEBI or GO for other types of entities.
- Clicking an edge allows making a request for supporting evidence collected from the literature and pathway databases in INDRA DB. The same panel also allows examining each INDRA Statement corresponding to the edge in more detail by clicking on “more info” under the “INDRA Statement details” heading.
- The force-directed active layout which snaps nodes to their positions can be disabled by toggling the “Forces” button.

The page initially displays a pre-built model which demonstrates all of these features. The RAS Pathway Map model was drawn by Dr. Frank McCormick in collaboration with the NCI RAS Initiative community.

Building models from text

Users have the ability to define their own biological models in natural language (English language text) under the “Build” tab. Here, we start off with the text necessary to build The NCI RAS Pathway Map as an example. A full list of the mechanistic relationships that can be represented by INDRA (and therefore INDRA-IPM) can be found at https://indra.readthedocs.io/en/latest/modules/statements.html, and examples of models described in natural language (processed via the TRIPS system and assembled by INDRA) can be found in Gyori, Bachman, et. al. (2017).

Users should note that the natural language processing systems are fairly robust but not without limitations. Proper grammar and punctuation should be used. The reading systems do not consider newlines to be sentence separators and may return erroneous output for sentences which are not terminated with a period.

The recognition and grounding of named entities (proteins, etc.) to database identifiers is done automatically. Nevertheless, using standardized names such as HGNC symbols (as opposed to informal synonyms) is preferred to avoid ambiguity. To normalize node names in the pathway map, the IPM performs name standardization, in which entities mentioned by their synonyms are normalized to standard names such as HGNC symbols (for instance, MEK1, Map2k1 and Mek1 are all normalized to the standard symbol MAP2K1). Note that by clicking on a node, a tooltip opens that allows linking out to databases (HGNC, UniProt, CiteAb), and checking the original text that the standardized node was created from.

INDRA-IPM also recognizes protein families and complexes and grounds them in the FamPlex ontology (https://github.com/sorgerlab/famplex/). In some cases, there is ambiguity in the name of a specific gene and a family it is part of. An example of this is the grounding of “JUN” from text to the JUN family, which also includes the JUN gene. In this case the user can use a synonym such as “c-JUN” that refers to the singular entity in order to reference only the gene and not the family.

We have exposed two reading systems to users. The REACH reader developed by the CLU Lab at the University of Arizona (https://github.com/clulab/reach) is an information extraction system for the biomedical domain, which aims to read scientific literature and extract cancer signaling pathways. We recommend users try REACH first due to its speed. The TRIPS/DRUM system (http://trips.ihmc.us/parser/cgi/drum) developed by IHMC may offer greater mechanistic detail in some use cases (for instance, it supports recognizing complex molecular conditions such as “BRAF-V600E not bound to Vemurafenib”), but it requires significantly longer to run.

Here is an example of a natural language model of the MAPK pathway that makes use of detailed mechanistic relations:

- Farnesylated KRAS translocates to the membrane.
- Active SOS1 activates KRAS that is at the membrane.
- Active RASA1 inhibits KRAS that is at the membrane.
- BRAF binds active KRAS.
- BRAF bound to KRAS is phosphorylated.
- Phosphorylated BRAF is active.
- Active BRAF phosphorylates MAP2K1 at S218 and S222.
- MAP2K1 phosphorylated at S218 and S222 is active.
- Active MAP2K1 phosphorylates MAPK1 at T185 and Y187.
- MAPK1 phosphorylated at T185 and Y187 is active.
- Active MAPK1 phosphorylates ELK1 at S383 and S389.
- ELK1 phosphorylated at S383 and S389 is transcriptionally active.
- Active MAPK1 phosphorylates ETS1 at T38.
- ELK1 translocates to the nucleus.
- ELK1 increases FOS.

- PPP3CA dephosphorylates ELK1 at S383.
- FOS binds to JUN.
- The FOS-JUN complex increases CCND1.
- CDK4 bound to CCND1 phosphorylates RB1 at S807.
- Active MAPK1 phosphorylates RPS6KA1 at T573.

Contextualizing Models

Users are able to project data from the Cancer Cell Line Encyclopedia (CCLE) onto their pathway maps. This is done automatically when the IPM is loaded initially (using the LOXIMVI skin cancer cell line) and can be changed to any other CCLE cell line in the Model Options dialogue panel. Wild type genes are colored green, while mutated genes are colored orange. Color intensity indicates the relative level of expression (see also the legend below the model canvas). Context is unavailable for gray nodes because they were not measured in CCLE.

Sharing Models

Users can share models using the NDEx network sharing website (http://ndexbio.org). To upload the current model, click the "NDEX" button at the bottom of the interface, then click "Upload". A link to NDEx will appear one the upload is complete.

One can load a model by entering the unique key at the end of this link (e.g., 9b901d8f-2e2d-11e9-9f06-0ac135e8bacf) into the Load field. Alternatively, one can share the link in the address bar (e.g., pathwaymap.indra.bio/?uuid=9b901d8f-2e2d-11e9-9f06-0ac135e8bacf) which will send a user to the IPM website and immediately load the shared model. Shared models preserve their text description, INDRA statements, graph layout, cell line context, and any evidence retrieved from INDRA DB.

Exporting Models

Users can export models in a variety of formats.

- INDRA JSON will export the model statements as a in the JSON format. These can be imported into INDRA or processed separately. The INDRA JSON format is specified at https://github.com/sorgerlab/indra/blob/master/indra/resources/statements_schema.json
- PySB, SBML, BNGL, Kappa will export executable models in these formats. These modeling formalisms allow parameterizing and simulating models, and evaluating them against time-course data. Additionally, the Kappa IM option downloads an image of the rule-based model's influence structure. More information about these formats and tools supporting them is available at the following places:
    - PySB: http://pysb.org/
    - SBML: http://sbml.org/
    - BNGL: http://visualizlab.org/rulebender/index.html and https://www.csb.pitt.edu/Faculty/Faeder/?page_id=409
    - Kappa: https://kappalanguage.org/
- SBGN will export a model in the Systems Biology Graphical Notation format. Documentation and tools supporting SBGN are available at: http://sbgn.github.io/sbgn/.

Note that layout information is not included in exported SBGN models, however tools such as Newt (http://web.newteditor.org/) have built-in layout algorithms.

- CX will export a model in the .cx format which can be opened in Cytoscape3 and also uploaded to NDEx.
    - Cytoscape enables network visualization and provides access to a large ecosystem of analysis plugins; more information is available at: https://cytoscape.org/cy3.html
    - NDEx is a network sharing and versioning website with a programmatic API for accessing networks: http://ndexbio.org/
- PNG will export a high-resolution .png image of the current graph. This feature is useful for taking snapshots of a pathway map for inclusion into documents or presentations.

In order to simplify the user interface, only PNG export is available on mobile devices with limited screen width.

Funding
This work was funded by ARO Grants W911NF-14-1-0397 and W911NF-15-1-0544 under the DARPA Big Mechanism and Communicating with Computers programs, and by NIGMS Grant P50GM107618.

Privacy

- Our API backend receives user-generated requests such as those for reading, contextualization, and NDEx sharing.
- Our server logs the IP addresses which make requests to the API.
- The data from some user requests is forwarded to external APIs such as TRIPS (reading), cBioPortal (contextualization), NDEx in order to implement these functions.
